# Supplementary material for: A Comparison of Spatial and Phenotypic Immune Profiles of Pancreatic Ductal Adenocarcinoma and Its Precursor Lesions
Source: Int J Mol Sci. 2024 Mar 3;25(5):2953. doi: 10.3390/ijms25052953 (PMC10932200; doi:10.3390/ijms25052953)

Supplemental Materials

Table S1. Characteristics of the different pancreatic cancer precursor lesions and pancreatic adenocarcinoma.

	MCN	IPMN	PanIN	PDAC
**Cystic**	yes	yes	no	no
**Location**	body/tail>head	head>body/tail	head=body=tail	head>body/tail
**Pancreatic duct communication**	no	yes	yes	yes
**Association with invasive cancer**	rarely	yes	yes	-
**Cytologic atypia**	low grade – high grade	low grade – high grade	low grade – high grade	-
**Molecular mutations**	KRAS, SMAD4, TP53	GNAS, KRAS, RNF43, TP53	KRAS, TP53, SMAD4/DPC4, BRCA2	KRAS, TP53, CDKN2A, SMAD4
**Age, decade**	4th-5th	6th-7th	6th-7th	7th-8th
**Sex predilection**	F>>>M	M>F	F>M	M>F

Figure S1. Composite images of multiplex fluorescent immunohistochemistry. A - F. Composite multispectral images of cystic lesions MCN (A) and IPMN (B), IPMN-associated PDAC (IAPA) (C), normal pancreatic tissue (NNPT) (D), PanIN (E), and PDAC (F) (original magnification, x20). White, ECs; green, CD4+ (helper) T cells; yellow, CD8+ (cytotoxic) T cells; red, Tregs; magenta, APCs.

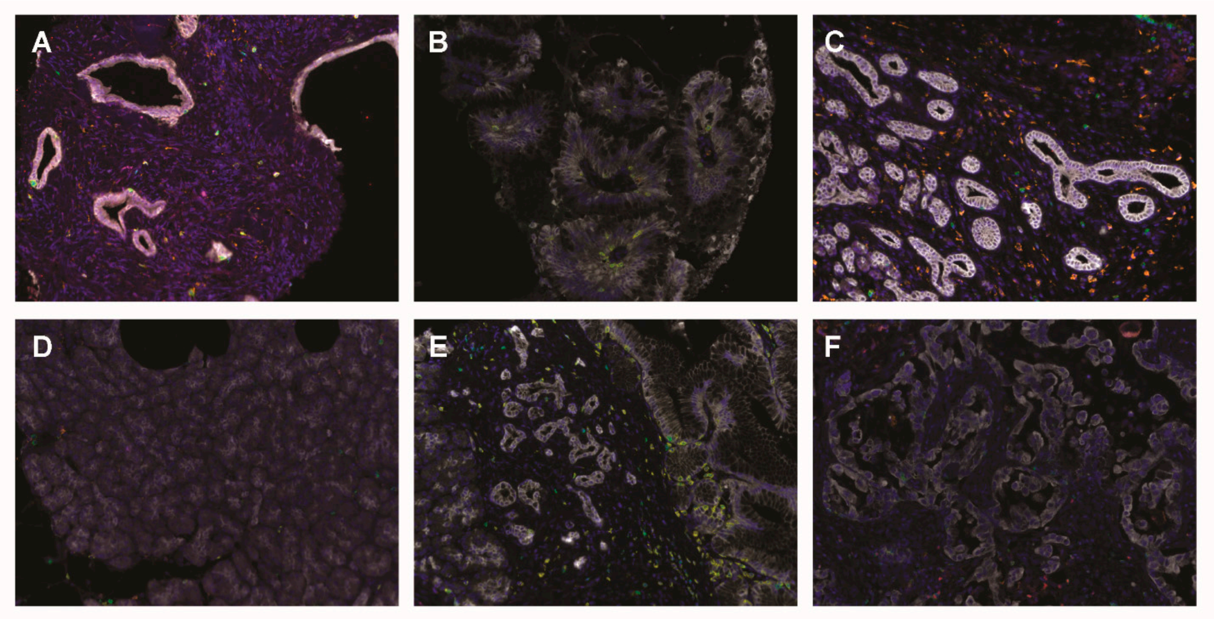

Figure S2. Composite image of PD-L1 staining (PD-L1+ cells) in PDAC.
White, ECs; green, CD4+ (helper) T cells; yellow, CD8+ (cytotoxic) T cells; red, Tregs; magenta, APCs; blue, PD-L1 positive cells (original magnification, x20).

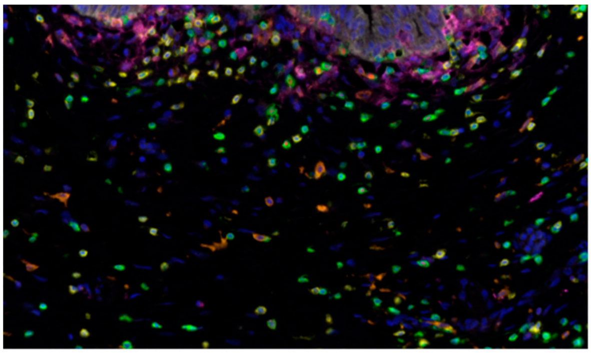

Figure S3. Schema depicting cells that are engaged or non-engaged. Cells are considered engaged if they are within a 15 μm radius from the center of a nucleus of a T cell or within a 40 μm radius from the center of a nucleus of another cell. If cells are outside that radius, they are considered non-engaged.

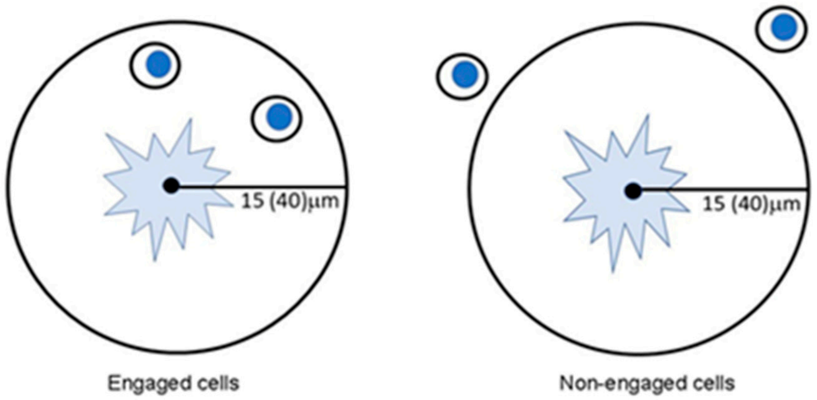

Figure S4. Engagement of CD8+ T cells with PD-L1 positive APCs in *cystic* lesions (A) and *non-cystic* lesions (C). Engagement of CD8+ T cells with PD-L1 positive ECs in *cystic* lesions (B) and *non-cystic* lesions (D). Normal pancreatic tissue used as a reference. * $p \leq 0.05$; ** $p \leq 0.01$; *** $p \leq 0.001$; **** $p \leq 0.0001$.

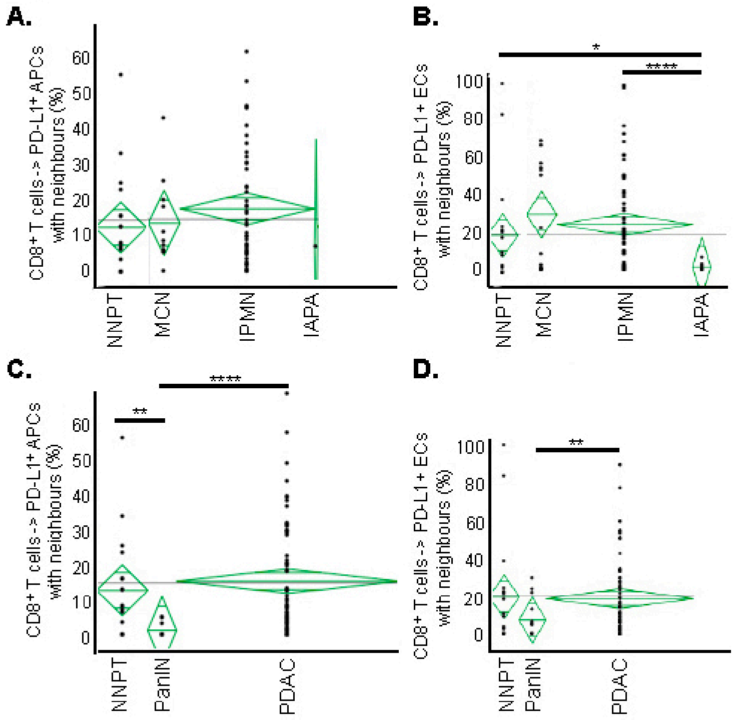

Table S2. Numbers of tissue samples of each tissue type used for the individual experiments.
Individual experiments are referred as to figures/images.

Figure	Image/Graph	NNPT	MCN	IPMN	IAPA	PanIN	PDAC
1	G, J	36	17	71	8	29	104
	H, K	36	17	71	8	29	104
	I, L	36	17	71	8	29	104
2	A, C	21	16	49	8	20	100
	B, D	21	16	49	8	20	100
3	A, C	36	17	71	8	29	106
	B, D	19	12	47	2	10	80
4	A, G	36	17	70	8	26	96
	B, H	36	15	66	8	27	98
	C, I	15	9	37	1	5	43
	D, J	11	8	35	8	13	75
	E, K	34	14	67	8	28	97
	F, L	36	15	71	8	29	93
5	A, C	36	14	72	8	28	104
	B, D	16	8	45	6	15	64
S4	A, C	14	11	44	1	4	69
	B, D	12	11	50	5	9	58

Figure S5. Neighbourhood analysis. The steps below represent the different steps we used to determine nearest neighbors. One representative example is shown.

First-order statistics
All cells
Number of cells: 2035
Average intensity: 0.006 cells per square micron
Frequency and intensity per phenotype:

	frequency	proportion	intensity
Cytotoxic T cells	217	0.107	0.001
Epithelial cells	346	0.170	0.001
APC	119	0.058	0.000
Other cell types	1299	0.638	0.004
T helper cells	4	0.002	0.000
T regs	50	0.025	0.000

Cell and phenotype locations:

1) Locations of all cells

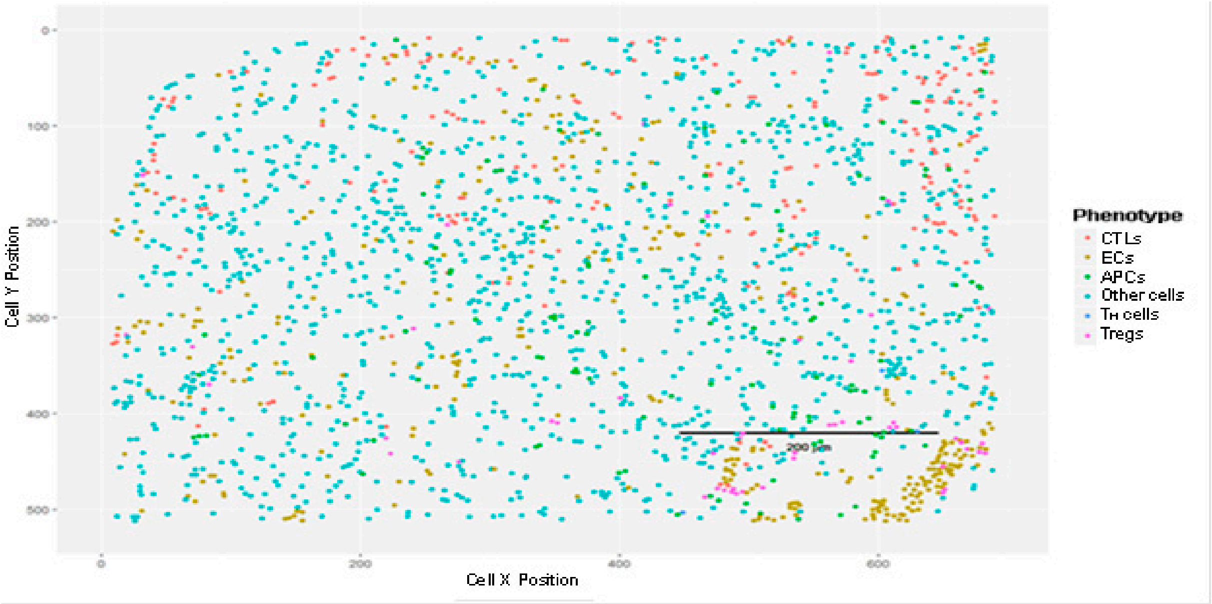

2) Individual cells

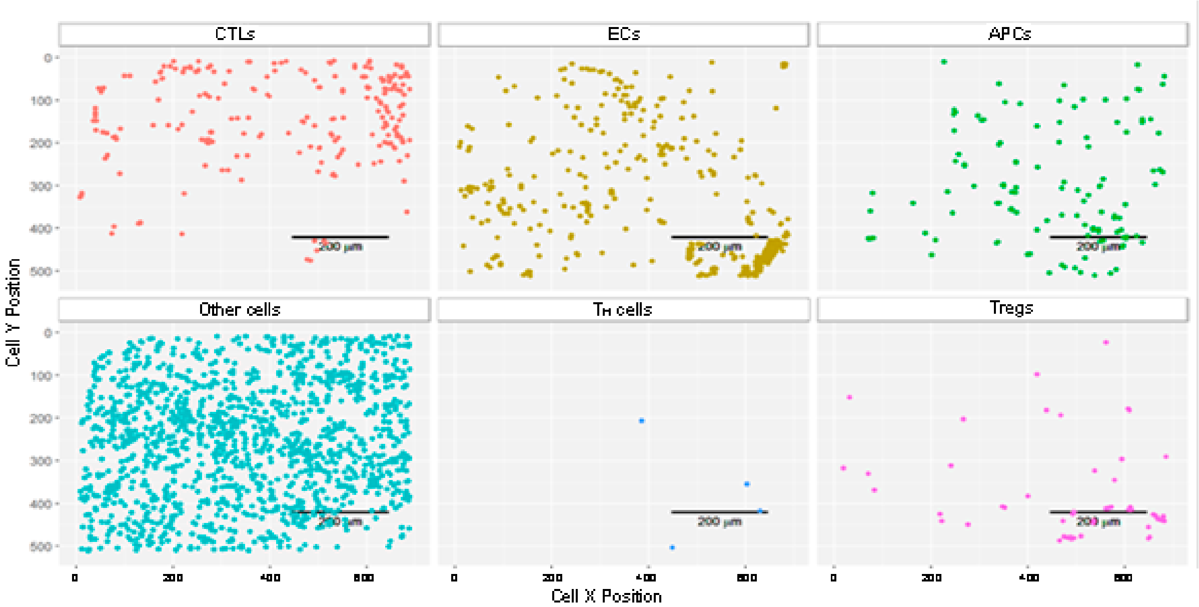

3) Nearest neighbours, selected phenotype

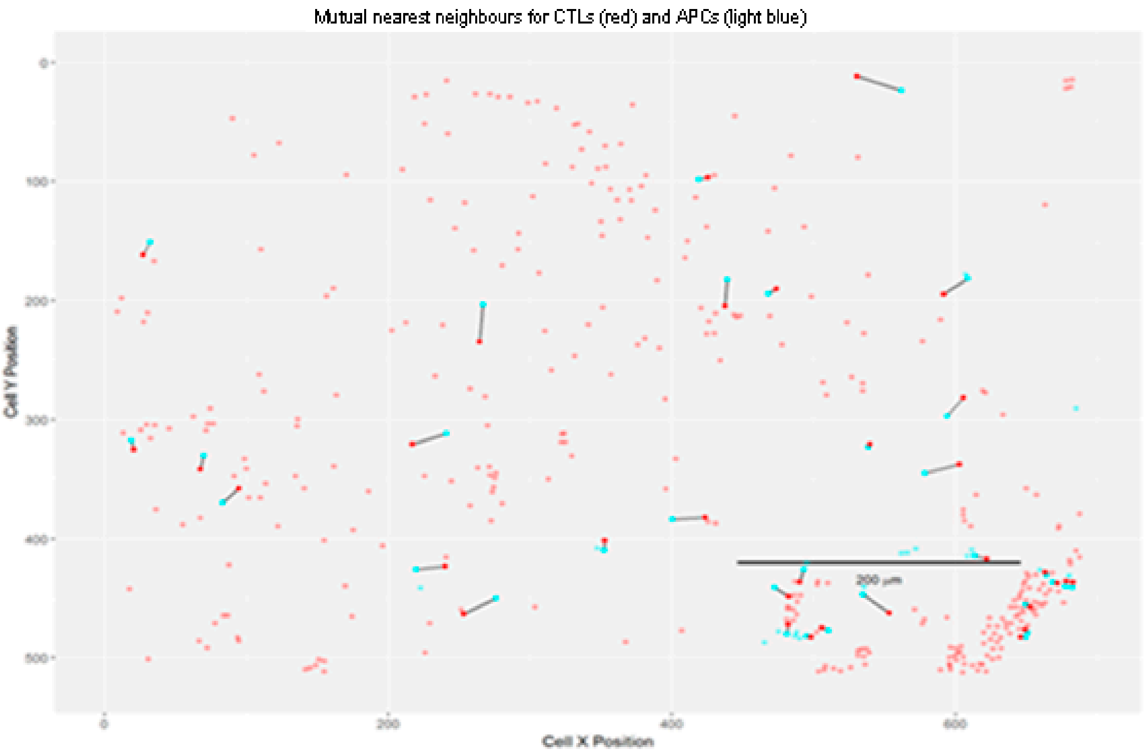

Supplement: Supplementary file 1 [file ijms-25-02953-s001.zip › ijms-2868847-supplementary.pdf]
